# Higher versus lower mean arterial blood pressure after cardiac arrest and resuscitation (MAP‐CARE): A protocol for a randomized clinical trial

**DOI:** 10.1111/aas.70040

**Published:** 2025-05-20

**Authors:** V. H. Niemelä, M. Reinikainen, N. Nielsen, F. Bass, P. Young, G. Lilja, J. Dankiewicz, N. Hammond, J. Hästbacka, H. Levin, M. Moseby‐Knappe, M. Saxena, M. Tiainen, A. Ceric, J. Holgersson, C. B. Kamp, J. Tirkkonen, T. Oksanen, T. Kaakinen, S. Bendel, J. Düring, A. Lybeck, J. Johnsson, J. Unden, A. Lundin, J. Kåhlin, J. Grip, E. Lotman, L. Romundstad, P. Seidel, P. Stammet, T. Graf, A. Mengel, C. Leithner, J. Nee, P. Druwé, K. Ameloot, M. P. Wise, P. J. McGuigan, J. White, M. Govier, M. Maccaroni, M. Ostermann, P. Hopkins, A. Proudfoot, R. Handslip, D. Pogson, P. Jackson, A. Nichol, M. Haenggi, M. P. Hilty, M. Iten, C. Schrag, M. Nafi, M. Joannidis, C. Robba, T. Pellis, J. Belohlavek, D. Rob, Y. Arabi, S. Buabbas, C. Yew Woon, A. Aneman, A. Stewart, C. Arnott, M. Ramanan, R. Panwar, A. Delaney, M. Reade, B. Venkatesh, L. Navarra, B. Crichton, D. Knight, A. Williams, H. Friberg, T. Cronberg, J. C. Jakobsen, M. B. Skrifvars

**Affiliations:** ^1^ Department of Anaesthesia and Intensive Care Helsinki University Hospital and University of Helsinki Helsinki Finland; ^2^ Institute of Clinical Medicine University of Eastern Finland Kuopio Finland; ^3^ Department of Anaesthesiology and Intensive Care Kuopio University Hospital Kuopio Finland; ^4^ Department of Clinical Sciences Lund, Anesthesia and Intensive Care Lund University Lund Sweden; ^5^ Department of Anesthesia and Intensive Care Helsingborg Hospital Helsingborg Sweden; ^6^ The George Institute for Global Health Sydney Australia; ^7^ Royal North Shore Hospital Sydney Australia; ^8^ Intensive Care Unit Wellington Hospital Wellington New Zealand; ^9^ Medical Research Institute of New Zealand Wellington New Zealand; ^10^ Australian and New Zealand Intensive Care Research Centre Monash University Melbourne Victoria Australia; ^11^ Department of Critical Care University of Melbourne Melbourne Victoria Australia; ^12^ Neurology, Department of Clinical Sciences Lund Lund University Lund Sweden; ^13^ Department of Neurology Skåne University Hospital Lund Sweden; ^14^ Department of Clinical Sciences Lund, Section of Cardiology Skåne University Hospital Lund Sweden; ^15^ Critical Care Program, The George Institute for Global Health UNSW Sydney Australia; ^16^ Malcolm Fisher Department of Intensive Care Royal North Shore Hospital Sydney New South Wales Australia; ^17^ Wellbeing Services County of Pirkanmaa and Tampere University, Faculty of Medicine and Health Technology Tampere University Hospital Tampere Finland; ^18^ Department of Clinical Sciences Lund Lund University Lund Sweden; ^19^ Department of Research, Development, Education and Innovation Skåne University Hospital Lund Sweden; ^20^ Department of Neurology and Rehabilitation Skåne University Hospital Lund Sweden; ^21^ Critical Care Division, Department of Intensive Care Medicine The George Institute for Global Health Sydney Australia; ^22^ St George Hospital Clinical School University of New South Wales Sydney New South Wales Australia; ^23^ Department of Neurology Helsinki University Hospital and University of Helsinki Helsinki Finland; ^24^ Anesthesia and Intensive Care, Department of Clinical Sciences Lund University, Skane University Hospital Malmö Sweden; ^25^ Copenhagen Trial Unit, Centre for Clinical Intervention Research Copenhagen University Hospital—Rigshospitalet Copenhagen Denmark; ^26^ Department of Regional Health Research, Faculty of Health Sciences University of Southern Denmark Copenhagen Denmark; ^27^ Intensive Care Unit Tampere University Hospital Tampere Finland; ^28^ Department of Anaesthesia and Intensive Care, Jorvi Hospital University Hospital of Helsinki and University of Helsinki Helsinki Finland; ^29^ Research Unit of Translational Medicine, Research Group of Anaesthesiology, Medical Research Center Oulu Oulu University Hospital and University of Oulu Oulu Finland; ^30^ OYS Heart, Oulu University Hospital MRC Oulu and University of Oulu Oulu Finland; ^31^ Department of Clinical Sciences, Anesthesia and Intensive Care Lund University, Skåne University Hospital Malmö Sweden; ^32^ Anesthesia and Intensive Care, Department of Clinical Sciences Lund Lund University, Skane University Hospital Lund Sweden; ^33^ Department of Operation and Intensive Care Hallands Hospital Halmstad Halmstad Sweden; ^34^ Department of Intensive and Perioperative Care, Skåne University Hospital Lund University Lund Sweden; ^35^ Department of Anaesthesiology and Intensive Care Medicine, Institute of Clinical Sciences, Sahlgrenska Academy University of Gothenburg Gothenburg Sweden; ^36^ Perioperative Medicine and Intensive Care (PMI) Karolinska University Hospital Stockholm Sweden; ^37^ Department of Physiology and Pharmacology Karolinska Institutet Stockholm Sweden; ^38^ Perioperative Medicine and Intensive Care Karolinska University Hospital Stockholm Sweden; ^39^ Department of Clinical Science, Intervention and Technology Karolinska Institute Stockholm Sweden; ^40^ North Estonia Medical Centre Tallinn Estonia; ^41^ Department of Anesthesia and Intensive Care Medicine, Division of Emergencies and Critical care Oslo University Hospital Oslo Norway; ^42^ Lovisenberg Diaconal University College Oslo Norway; ^43^ Department of Intensive Care Medicine Stavanger University Hospital Stavanger Norway; ^44^ Department of Anaesthesia and Intensive Care Medicine Centre Hospitalier de Luxembourg Luxembourg Luxembourg; ^45^ Department of Life Sciences and Medicine, Faculty of Science, Technology and Medicine University of Luxembourg Esch‐sur‐Alzette Luxembourg; ^46^ University Hospital Schleswig‐Holstein University Heart Center Lübeck Lübeck Germany; ^47^ German Center for Cardiovascular Research (DZHK), partner site Hamburg/Lübeck/Kiel Germany; ^48^ Department of Neurology and Stroke University Hospital Tuebingen Tuebingen Germany; ^49^ Hertie Institute of Clinical Brain Research Tuebingen Germany; ^50^ Department of Neurology Freie Universität and Humboldt‐Universität zu Berlin, Charité—Universitätsmedizin Berlin Berlin Germany; ^51^ Department of Nephrology and Medical Intensive Care Charité—Universitaetsmedizin Berlin Berlin Germany; ^52^ Department of Intensive Care Medicine Ghent University Hospital Ghent Belgium; ^53^ Department of Cardiology Ziekenhuis Oost‐Limburg Genk Belgium; ^54^ Adult Critical Care University Hospital of Wales Cardiff UK; ^55^ Wellcome‐Wolfson Institute for Experimental Medicine Queen's University Belfast Belfast UK; ^56^ Regional Intensive Care Unit Royal Victoria Hospital Belfast UK; ^57^ CEDAR (Centre for Healthcare Evaluation, Device Assessment and Research) Cardiff and Vale University Health Board Cardiff Cardiff UK; ^58^ Bristol Royal Infirmary University Hospitals Bristol and Weston Bristol UK; ^59^ Essex Cardiothoracic Centre Essex UK; ^60^ Anglia Ruskin School of Medicine ARU Essex UK; ^61^ Guy's & St Thomas' Hospital London UK; ^62^ Intensive Care Medicine Centre for Human & Applied Physiological Sciences, School of Basic & Medical Biosciences, Faculty of Life Sciences Medicine King's College London UK; ^63^ Intensive Care Medicine, King's Critical Care King's College Hospital, NHS Foundation Trust London UK; ^64^ Department of Perioperative Medicine, Barts Heart Centre St Bartholomew's Hospital London UK; ^65^ St George's University Hospital NHS Foundation Trust London UK; ^66^ Department of Critical Care Portsmouth University Hospitals Trust Cosham Portsmouth UK; ^67^ Leeds Teaching Hospitals NHS Trust Leeds UK; ^68^ University College Dublin Clinical Research Centre at St Vincent's University Hospital University College Dublin Dublin Ireland; ^69^ The Alfred Hospital Melbourne Australia; ^70^ Institute of Intensive Care Medicine University Hospital Zurich Zurich Switzerland; ^71^ Department of Intensive Care Medicine Inselspital University Hospital Bern Bern Switzerland; ^72^ Klinik für Intensivmedizin Kantonsspital St. Gallen St. Gallen Switzerland; ^73^ Istituto Cardiocentro Ticino Lugano Switzerland; ^74^ Division of Intensive Care and Emergency Medicine, Department of Internal Medicine Medical University Insbruck Innsbruck Austria; ^75^ IRCCS Policlinico San Martino Genoa Italy; ^76^ Dipartimento di Scienze Chirurgiche Diagnostiche Integrate University of Genova Genova Italy; ^77^ Anaesthesia and Intensive Care Pordenone Hospital Azienda Sanitaria Friuli Occidentale Pordenone Italy; ^78^ 2nd Department of Internal Medicine, Cardiovascular Medicine General University Hospital, 1st Faculty of Medicine, Charles University in Prague Prague Czech Republic; ^79^ Institute for Heart Diseases Wroclaw Medical University Wrocław Poland; ^80^ 2nd Department of Medicine, Department of Cardiovascular Medicine, First Faculty of Medicine Charles University in Prague, General University Hospital in Prague Prague Czech Republic; ^81^ King Saud bin Abdulaziz University for Health Sciences and King Abdullah International Medical Research Center Riyadh Saudi Arabia; ^82^ Department of Anesthesia, Critical Care and Pain Medicine Jaber Alahmad Alsabah Hospital Kuwait; ^83^ Tan Tock Seng Hospital Singapore Singapore; ^84^ Yong Loo Lin School of Medicine National University of Singapore Singapore Singapore; ^85^ Lee Kong Chian School of Medicine Nanyang Technological University Singapore Singapore; ^86^ Intensive Care Unit Liverpool Hospital, South Western Sydney Local Health District Sydney New South Wales Australia; ^87^ South Western Clinical School University of New South Wales Sydney New South Wales Australia; ^88^ The Ingham Institute for Applied Medical Research Sydney New South Wales Australia; ^89^ Liverpool Hospital Sydney New South Wales Australia; ^90^ Department of Cardiology Royal Prince Alfred Hospital Sydney New South Wales Australia; ^91^ Caboolture and Royal Brisbane and Women's Hospitals Metro North Hospital and Health Service Brisbane Queensland Australia; ^92^ School of Clinical Medicine Queensland University of Technology Brisbane Queensland Australia; ^93^ Critical Care Division, The George Institute for Global Health University of New South Wales Sydney New South Wales Australia; ^94^ School of Medicine and Public Health University of Newcastle Newcastle New South Wales Australia; ^95^ Intensive Care Unit John Hunter Hospital Newcastle New South Wales Australia; ^96^ Northern Clinical School, Sydney Medical School University of Sydney Sydney New South Wales Australia; ^97^ Department of Intensive Care Christchurch Hospital Christchurch New Zealand; ^98^ Middlemore Hospital Auckland New Zealand; ^99^ Anesthesia and Intensive Care, Department of Clinical Sciences Lund Lund University Lund Sweden; ^100^ Intensive and Perioperative Care Skåne University Hospital Malmö Sweden

**Keywords:** blood pressure, cardiac arrest, randomized clinical trial, target

## Abstract

**Background:**

In patients resuscitated after cardiac arrest, a higher mean arterial pressure (MAP) may increase cerebral perfusion and attenuate hypoxic brain injury. Here we present the protocol of the mean arterial pressure after cardiac arrest and resuscitation (MAP‐CARE) trial aiming to investigate the influence of MAP targets on patient outcomes.

**Methods:**

MAP‐CARE is one component of the Sedation, Temperature and Pressure after Cardiac Arrest and Resuscitation (STEPCARE) 2 x 2 x 2 factorial randomized trial. The MAP‐CARE trial is an international, multicenter, parallel‐group, investigator‐initiated, superiority trial designed to test the hypothesis that targeting a higher (>85 mmHg) (*intervention*) versus a lower (>65 mmHg) (*comparator*) MAP after resuscitation from cardiac arrest reduces 6‐month mortality (*primary outcome*). Trial participants are adults with sustained return of spontaneous circulation who are comatose following resuscitation from out‐of‐hospital cardiac arrest. The two other components of the STEPCARE trial evaluate sedation and temperature control strategies. Apart from the STEPCARE trial interventions, all other aspects of general intensive care will be according to the local practices of the participating site. Neurological prognostication will be performed according to European Resuscitation Council and European Society of Intensive Care Medicine guidelines by a physician blinded to allocation group. The sample size of 3500 participants provides 90% power with an alpha of 0.05 to detect a 5.6 absolute risk reduction in 6‐month mortality, assuming a mortality of 60% in the control group. Secondary outcomes will be poor functional outcome 6 months after randomization, patient‐reported overall health 6 months after randomization, and the proportion of participants with predefined severe adverse events.

**Conclusion:**

The MAP‐CARE trial will investigate if targeting a higher MAP compared to a lower MAP during intensive care of adults who are comatose following resuscitation from out‐of‐hospital cardiac arrest reduces 6‐month mortality.

## 1 BACKGROUND

Maintaining cerebral perfusion is an essential part of post‐cardiac arrest care.^1^ Several studies have shown an association between hypotension and poor outcome.^2^, ^3^, ^4^, ^5^, ^6^, ^7^, ^8^, ^9^, ^10^ Currently, the American Heart Association recommends a mean arterial pressure (MAP) target >80 mmHg unless the patient has invasive brain monitoring in place.^11^ In contrast, the European Resuscitation Council (ERC) recommends targeting a MAP >65 mmHg, a urine output of >0.5 mL/kg/h, and a normal or decreasing serum lactate.^12^ Maintaining a higher MAP compared to a lower MAP after cardiac arrest and resuscitation may increase perfusion of the brain, heart, and other organs and thus improve patient outcomes.^10^, ^13^

Four trials have randomized patients to a higher or lower MAP target after cardiac arrest. The meta‐analysis of these trials did not find a difference in mortality or functional outcome between blood pressure target groups.^14^ The blood pressure and Oxygenations Targets in Post Resuscitation Care trial^15^ has the highest methodological quality. The trial compared a higher MAP target of 77 mmHg to a lower MAP target of 63 mmHg after cardiac arrest and did not find a difference in the composite outcome of death or poor functional outcome at 3 months.^15^ The strength of this trial is its blinded design, but important limitations include randomizing only patients with a cardiac cause of the arrest, a sample size that cannot exclude realistic treatment effects, and a relatively small difference in achieved blood pressures between groups (10–15 mmHg). The results of this trial may not be generalizable because it was conducted in only two centers within the same country and observed mortality was lower than that reported in most cardiac arrest trials.^15^, ^16^, ^17^, ^18^

Two smaller feasibility trials^19^, ^20^ investigating blood pressure targets after cardiac arrest did not demonstrate a difference in biomarker neuron‐specific enolase (NSE) levels that were used as a surrogate outcome measure of brain injury. However, the higher MAP target was associated with lower levels of another brain injury biomarker, neurofilament light (NfL), in a post hoc analysis.^21^ The higher MAP target was associated with lower levels of high‐sensitivity cardiac troponin‐T (hs‐cTnT) in a subpopulation of patients with acute myocardial infarction and shock.^22^

Maintaining a higher MAP compared to a lower MAP requires higher doses of vasoactive medication.^15^, ^19^, ^20^, ^23^ Vasopressors and inotropes have dose‐dependent adverse effects.^24^, ^25^ Thus, maintaining a higher MAP may predispose patients to cardiac arrhythmias, non‐occlusive mesenteric ischemia, limb ischemia, and bleeding complications.^26^, ^27^, ^28^, ^29^, ^30^, ^31^ However, a meta‐analysis of the randomized clinical trials of higher versus lower blood pressure targets did not demonstrate a difference in adverse events between MAP target groups.^14^

Here, we describe the mean arterial pressure after cardiac arrest and resuscitation (MAP‐CARE) trial which is part of the factorial Sedation, Temperature, and Pressure after Cardiac Arrest and Resuscitation (STEPCARE) trial. The two other interventions of the STEPCARE factorial trial (sedation and temperature) are described separately.

## 2 METHODS

### 2.1 Trial design

The MAP‐CARE trial is registered at clinicaltrials.gov (NCT05564754, 2022‐10‐03) as part of the 2 x 2 x 2 factorial STEPCARE trial. The STEPCARE trial protocol was designed following the standard protocol items: recommendations for interventional trials guidelines,^32^ and the trial will be reported according to the CONSORT guidelines.^33^, ^34^ The full STEPCARE trial protocol is available at www.stepcare.org. The STEPCARE trial is a randomized international, multicenter, parallel‐group, investigator‐initiated, superiority trial with three simultaneous intervention arms, considered three separate trials. In the MAP‐CARE trial, participants will be randomized to higher or lower MAP target groups. In the two other interventions, participants will be randomized to continuous deep sedation or minimal sedation and fever treatment with or without a device. All participants will be randomized to all three interventions of the STEPCARE trial; thus, selective participation is not possible. Apart from the interventions of the STEPCARE trial, intensive care management will be according to the international guidelines and local practices of each participating hospital.

### 2.2 Inclusion criteria

MAP‐CARE will include adults (≥18 years) who experience an out‐of‐hospital cardiac arrest (OHCA) with sustained return of spontaneous circulation (ROSC; 20 min of spontaneous circulation without the need for chest compressions) and who are unconscious, defined as not being able to obey verbal commands (Full Outline of UnResponsiveness [FOUR] score motor response <4),^35^ or who are intubated and sedated because of agitation after sustained ROSC. Participants must not have treatment limitations for intensive care (e.g., a “do not attempt resuscitation” order or a decision not to escalate care) to be included in the trial. Screening will be performed as soon as possible but no later than 240 min after ROSC.

### 2.3 Exclusion criteria

Exclusion criteria will be trauma or hemorrhage (including gastrointestinal bleeding) as the presumed cause of the arrest, suspected or confirmed intracranial hemorrhage, extracorporeal membrane oxygenation (ECMO) prior to randomization, pregnancy, and previous randomization to the STEPCARE trial.

### 2.4 Screening and randomization

Screening will be performed in the emergency room, angiography suite, or the intensive care unit (ICU). Clinical investigators at each participating site will be responsible for screening all patients resuscitated from an OHCA. A screening log will be compiled, including all cardiac arrest patients with sustained ROSC admitted to the ICU, to document whether they are eligible for inclusion. Informed consent will be obtained according to national ethical approvals. The reason for the exclusion of screened patients will be documented and reported. Randomization will be performed via a web‐based application to allow immediate allocation to treatment groups and ensure allocation concealment and adequate allocation sequence generation. Randomization will be performed with blinded permuted blocks of varying size, stratified for trial site.

### 2.5 Intervention

Participants will be randomly assigned to a MAP target of either >85 or >65 mmHg. All participants must have invasive blood pressure monitoring. After randomization, adjusting vasoactive medications and fluid therapy to achieve the MAP target will begin as soon as possible. If the MAP is higher than the allocated target and the patient is on vasopressors, then the vasopressor should be titrated down to achieve the target. There is no need to lower MAP if it is above the MAP target, but the patient is not receiving vasopressors. Hypertensive urgencies occurring in the absence of vasoactive medications will be managed in accordance with usual practice in both the low MAP and high MAP arms.

Treating clinicians will determine the methods to achieve the MAP target, but the primary recommendation is to titrate a vasopressor unless the patient is hypovolemic. Fluid therapy should follow standard procedures. If hypovolemia is suspected and the patient is fluid‐responsive, a fluid bolus may be given. However, excessive fluid loading should be avoided.^36^

The intervention continues for up to 72 h after randomization or until extubation, whichever occurs first. After this period, and after ICU discharge, MAP management will be at the treating physician's discretion. The allocated MAP target will be reinstated if a participant is reintubated during the 72‐h intervention period.

The aim is to maintain the MAP target throughout as much of the intervention period as possible, but adjustments might be necessary based on the clinical situation. If needed, MAP target adjustments should be made in 5 mmHg increments or decrements. Reasons for deviations from the allocated MAP target will be recorded (Supporting Information S1; Table S1). If the clinical situation changes, efforts should be made to achieve the allocated MAP target.

### 2.6 General Intensive care

General intensive care, including management of respiration, metabolic disturbances, ulcer prophylaxis, deep venous thrombosis prophylaxis, and other aspects of intensive care, should be delivered similarly in all allocation groups and according to local protocols at the discretion of the treating physicians. Cardiac interventions will also be guided by local protocols. However, participating centers will need to have access to around‐the‐clock invasive management, either on‐site or at a nearby hospital, which is also part of the trial. Cardiac catheterization (coronary angiography) should not be delayed by the trial interventions. Apart from the interventions, adhering to international and national guidelines for post‐resuscitation care is recommended.

### 2.7 Blinding

The clinical team responsible for the immediate care of the participant will not be blinded to the study interventions due to inherent difficulty in blinding the interventions (sedation, temperature, and blood pressure). Measures will be taken to ensure that allocation information will be disseminated only within the immediate group of healthcare workers responsible for patient care. A blinded physician will make a first prognostic evaluation of the participant 72 h after randomization and make a statement on neurological prognosis (for details, see below).

Participants, their legal representatives, and family will only be informed that the patient has been part of the trial, but not the allocation group. The outcome assessors, prognosticators, statisticians, the data and safety monitoring committee (DSMC), members of the steering group, and authors of the manuscript will be blinded to treatment allocation. The intervention groups will be coded as “X” and “Y.” Two abstracts will be prepared, one assuming X is the experimental group and Y is the comparator group, and one assuming the opposite. The author group must approve conclusions before the code is broken.

### 2.8 Prognostication and withdrawal of life‐sustaining therapies

The MAP‐CARE trial will employ a conservative and strict protocol for neurological prognostication according to the ERC and the European Society of Intensive Care Medicine (ESICM) recommendations (see Supporting Information S1).^12^, ^37^ Prognostication will be performed on all participants who are not awake and obeying verbal commands, and who are still in the ICU at 72 h after randomization. Prognostication must be sufficiently delayed, ensuring that any lingering effects of sedative agents will not affect the assessment. Prognostication will be made by a physician experienced in neuro‐prognostication after cardiac arrest and blinded to treatment allocations. The blinded external physician will not make specific recommendations about withdrawal of life‐sustaining measures.

Presumed poor functional outcome will not justify the withdrawal of life‐sustaining therapies (WLST) prior to prognostication. Life‐sustaining therapies may only be withdrawn before protocolized prognostication in the following situations: information on a pre‐existing advanced care directive or an advanced medical comorbidity (e.g., generalized malignant disease) that prohibits continuation of care becomes available after inclusion in the trial or continuation of care is considered unethical due to irreversible multi‐organ failure. Brain death, established according to local legislation, will be defined as death and not WLST. See Supporting Information S1 for a detailed description of neurological prognostication and WLST.

### 2.9 Follow‐up

Long‐term outcomes will be assessed and recorded during a telephone follow‐up at 30 days and during a physical visit or a telephone/virtual meeting 6 months after randomization. The blinded outcome assessor may be an occupational therapist, physician, research nurse, psychologist, or another health care professional. The central follow‐up coordinating team will provide outcome assessors with detailed guidelines and study‐specific training. More detailed outcomes will be assessed in an extended follow‐up sub‐study at selected sites, including, for example, cognitive function, societal participation, and family impact. This sub‐study is described elsewhere.

### 2.10 Outcome measures

The primary and secondary outcomes will be assessed 6 months after randomization. The primary outcome will be all‐cause mortality. Secondary outcomes will be the proportion of participants with a poor functional outcome defined primarily as a score of 4–6 (moderately severe disability, severe disability, or death) reported by the structured modified Rankin Scale (mRS, range 0–6, with higher scores indicating a worse outcome). If an mRS score cannot be assigned, patients will be categorized based on whether they are dependent on others for basic activities of daily life (need of assistance with, e.g., moving indoors, eating, dressing, taking care of personal hygiene), similar to an mRS score of 4–6 but without the detailed information that is needed for precise scoring. Other secondary outcomes will include the proportion of patients who died or had a predefined serious adverse event in the ICU, and patient‐reported overall health by using the EuroQol (EQ) visual analog scale (EQ VAS, a part of the EQ‐5D).

Exploratory outcomes will be ventilator‐free days within the first 30 days, hospital‐free days within the first 30 days, mRS (ordinal score), time‐to‐event and win ratio (dead versus alive), all steps on the mRS scale, safety events, and detailed information from the EQ‐5D‐5L.

### 2.11 Adverse events

It is recognized that the intensive care patient population will experience several common aberrations in laboratory values, signs, and symptoms due to the severity of the underlying disease and the impact of standard therapies. Intensive care patients will frequently develop life‐threatening organ failure(s) unrelated to study interventions, despite optimal management. Therefore, consistent with established practice in academic ICU trials,^38^ events that are part of the natural history of the primary disease process or expected complications of critical illness will not be reported as adverse events in this study. All adverse events potentially causally related to the study intervention or that are of concern in the investigator's judgment will be reported and reviewed by the DSMC. Several specified severe adverse events (SAEs) are captured in the trial case report form and will not be separately reported as SAEs.

Only predefined SAEs (Table 1) and any unexpected SAE will be reported by the investigator to avoid overreporting and to maximize the probability of finding true and important differences. We predefined SAEs as potential harms from blood pressure interventions, based on a meta‐analysis of randomized controlled trials (RCTs) comparing MAP targets after cardiac arrest^14^ and an RCT comparing MAP targets in patients with septic shock.^40^ Based on these studies, the incidence of arrhythmia resulting in hemodynamic compromise is expected to be around 13%, the incidence of moderate to severe bleeding is expected to be 9%, the incidence of digital ischemia is expected to be 2%, the incidence of mesenteric ischemia is expected to be 2%, and the incidence of acute kidney injury (AKI) necessitating renal replacement therapy is expected to be 35%.^14^, ^40^

**TABLE 1:** Definition of specific serious adverse events reported as secondary outcomes.

Serious adverse event	Definition
Arrhythmia	Arrhythmia requiring defibrillation, cardioversion, or chest compressions
Moderate or severe bleeding	Intracerebral bleeding, bleeding resulting in substantial hemodynamic compromise requiring treatment or need for blood transfusion^39^
Acute kidney injury	Requiring renal replacement therapy
Limb or digital necrosis	Requiring radiological or surgical intervention
Gut ischemia	Verified by imaging, endoscopy, or requiring surgery

### 2.12 Rationale for chosen outcomes

All‐cause mortality was chosen as the primary outcome to ensure an unbiased assessment and to avoid competing risks. We will use the mRS to evaluate functional outcome. The mRS scale is increasingly used in cardiac arrest research and is currently recommended by the Core Outcome Set for Cardiac Arrest (COSCA) and the International Liaison Committee on Resuscitation (ILCOR) consensus statement for measuring functional outcome after cardiac arrest.^41^ The primary analysis will be a binary analysis, with the mRS dichotomized as 0–3 (none to moderate disability) versus 4–6 (moderately severe disability to death) as this dichotomization separates patients that are non‐dependent from patients that are dependent on others in basic activities of daily living. This dichotomization is also previously used in cardiac arrest trials.^41^ The EQ‐VAS included as a part of EQ‐5D‐5L will be used to measure a patient‐reported outcome of overall health status. This instrument was chosen since it is simple to use, with its validity supported by evidence, and can be based on a proxy report if necessary.^42^ We will measure possible harmful effects of the intervention by predefined SAEs that are most common and plausibly related to the intervention. A more detailed description of the rationale for chosen outcomes is available in Supporting Information S1.

### 2.13 Factorial design

Factorial trials have the inherent risk of potential interactions between interventions on both physiological and patient‐centered outcomes.^43^ This trial is conducted assuming no interaction between the interventions on specified patient‐centered outcomes. The MAP‐CARE trial intervention has potential physiological interactions with the sedation and temperature interventions of the STEPCARE trial. Targeting deep sedation may cause additional vasodilation and hypotension after cardiac arrest and, therefore, affect the achievement of the allocated MAP target. However, no evidence suggests these interactions could affect the assessed outcomes. If higher doses or levels of sedation, inotropic/vasopressor support, or external cooling are required because of between group interactions, differential adverse effects of these interactions are theoretically possible. The DSMC will monitor the trial during its conduct to identify possible interaction effects on outcomes, focusing on patient safety.

### 2.14 Co‐enrolment in other trials

Study participants may be included in any observational study which does not affect protocol adherence in the STEPCARE trial. We will assess co‐enrolment suggestions based on the Spice‐8 co‐enrolment guidelines.^44^ Unless there are clear conflicts between trial interventions, co‐enrolment in other trials will be possible. The STEPCARE management committee will assess co‐enrolment on a case‐by‐case basis.

### 2.15 Data collection and management

Individual patient data regarding background characteristics, clinical features, and laboratory results will be obtained from medical and ambulance service records and relatives. Detailed data including neurological status, body temperature, blood pressure values, and doses of vasoactive and sedative medications will be collected. Data will be entered into a web‐based electronic Case Report Form (eCRF) by site personnel. Detailed information on collected data is presented in the Supporting Information S1. The software for eCRF is provided by Spiral, New Zealand, but the storage server for the trial database is handled by the trial's coordinating team.

### 2.16 Sample size and power estimations

The sample size estimation is based on a 60% mortality in the control arm and a 54.4% mortality in the intervention arm at 6 months, referring to the results of the targeted temperature management (TTM) EuroQol (‐trial,^45^ TTM2‐trial^17^ and the international cardiac arrest registry (INTCAR).^46^ To demonstrate a relative risk of 0.91 with 90% power at a significance level of 0.05, using two‐sided tests, 1639 participants are required in each group, a total of 3278 participants. In the TTM2 trial,^17^ loss to follow‐up was approximately 2%, and we expect similar loss to follow‐up in the STEPCARE trial. Therefore, the sample size was increased by 6.8% to 3500 participants; 1.8% of the increment is considered to account for loss to follow‐up, and, as a pragmatic choice, 5% is considered to account for possible interactions between interventions on patient‐centered outcomes. The sample size calculation corresponds to a relative risk reduction of 9.3%, and an absolute risk reduction of 5.6%, which is a clinically relevant and realistic treatment effect. For the secondary outcomes, there is an estimated power of 91% to detect a relative risk reduction of 0.9 for poor outcome (mRS 4–6), a power of >90% to detect a difference of five points on the EQ‐5D‐5L VAS scale, and a power of 91% to detect a relative risk reduction or increase of 10% for the predefined serious adverse events (Table 1).

### 2.17 Statistical analyses

All analyses will be conducted according to the intention‐to‐treat principle and adjusted for site and the allocated intervention in the two other trials of the factorial STEPCARE trial. Dichotomous outcomes will be presented as proportions of participants with the event and relative risks with 95% confidence intervals. Continuous data will be presented as means and standard deviations for each group, with 95% confidence intervals for the means of the groups and the differences between the means of the groups. Count data will be presented as means, mean differences, and 95% confidence intervals or medians, interquartile ranges, and 95% confidence intervals depending on the observed distribution. Dichotomous outcomes will be analyzed using a mixed effects generalized linear model, continuous outcomes using a mixed effects linear regression model, and count data using the Wilcoxon test. Mock tables, curves, and graphs presenting characteristics of the participants, results, and separation of blood pressure and vasopressor dose between the groups are provided in the Supporting Information S1. A detailed statistical analysis plan will be published separately.

### 2.18 Subgroup analysis

The following subgroup analyses will be performed:

- Age (<median or ≥median)
- Sex (male/female)
- Bystander cardiopulmonary resuscitation (yes/no)
- Initial rhythm (shockable versus non‐shockable)
- Time to ROSC (<median or ≥median)
- Circulatory status on admission (presence or absence of circulatory shock diagnosed by the treating physician)
- Baseline risk of poor functional outcome (Miracle2‐score: low risk [0–2], medium risk [3–5], and high risk [6–10])^47^
- Presumed cause of cardiac arrest at randomization (cardiac vs. others), and
- Previous diagnosis of hypertension

### 2.19 Sub‐studies

The main sub‐studies of the STEPCARE trial include a biomarker study, an AKI study, a neuroprognostic study, intensive care monitoring studies, and an extended follow‐up study. Separate protocols will be published for these sub‐studies. Additional sub‐studies will be presented on the STEPCARE trial webpage (www.stepcare.org) and the protocols for these sub‐studies will be published separately.

### 2.20 Ethics and informed consent

Ethics application sought approval for a delayed written consent process, since the intervention must be regarded as an emergency procedure and must be started as soon as the participants are admitted to hospitals. Participants regaining consciousness will be asked for informed consent as soon as they are able to make an informed decision. It is of importance that ethical approval contains a request to use data also for deceased participants, to avoid survivor bias. The consent process will vary from site to site and align with ethics approval, national law, and the Declaration of Helsinki.

### 2.21 Data and safety monitoring and interim analysis

The Charter for the DSMC of the STEPCARE trial describes the role and function of the DSMC. The primary focus of the DSMC is monitoring the safety and efficacy of the interventions and the overall conduct of the trial to guard the interests of the trial participants. The first interim analysis was conducted after the enrollment of 500 participants, with the recommendation to *continue the trial as planned*. The schedule of further interim analyses will be decided by the DSMC, but a minimum of three interim analyses will be conducted. The DSMC will arrange for an independent statistician to conduct a blinded interim analysis. The DSMC can request unblinding of data if required. The survival and safety parameters are provided for the DSMC for the conduct of the interim analyses. Lan‐DeMets group sequential monitoring boundaries will be used as the statistical limit to guide recommendations regarding the early termination of the trial.^48^ Interventions of the STEPCARE trial will not be stopped for futility. The DSMC may recommend stopping or pausing the MAP‐CARE trial or the entire STEPCARE trial if:

- Between group difference in the primary outcome measure is found in the interim analysis according to predefined stopping rules;
- Between group difference in serious adverse events is found in the interim analysis;
- Evidence of interaction influencing outcomes; or
- Results from other studies show convincingly benefit or harm with one of the allocation arms.

It is the steering group's decision whether the trial should be stopped.

### 2.22 Patient group involvement

We followed the COSCA guidelines, developed in collaboration with ILCOR, which involved patient representatives to facilitate the selection of patient‐centered outcomes.^41^ Patient organizations in Sweden and Australia were involved in the design phase of the STEPCARE trial.

### 2.23 Trial status and timeline

Randomization began in August 2023 and trial sites have been added gradually. The last 6‐month follow‐up will be performed presumably during 2026–2027. Results from each intervention and sub‐study will be reported separately. The initial publications for each STEPCARE trial will include results of primary and secondary outcomes.

## 3 DISCUSSION

The aim of this trial is to investigate whether a higher, as compared to a lower, MAP target improves survival, functional outcome, and patient‐reported health status in a broad OHCA patient population. An additional aim is to assess whether the incidence of predefined serious adverse events differs between the MAP target groups. Results from the MAP‐CARE trial will guide future recommendations on post‐cardiac arrest blood pressure targets. The MAP‐CARE trial is part of the factorial STEPCARE trial that also investigates whether the depth of sedation and device‐based fever treatment affect recovery after cardiac arrest.

The optimal MAP target after cardiac arrest is yet to be established; therefore, further research is mandated.^49^, ^50^ Randomized clinical trials on MAP targets conducted so far have included mainly OHCA patients with a cardiac cause of arrest.^15^, ^19^, ^20^, ^51^ These patients generally have a better prognosis than patients with a cardiac arrest of different origin.^45^, ^52^ The STEPCARE trial has broadened inclusion criteria, also including patients with a hypoxic cardiac arrest origin. This approach allows us to investigate whether patients in this population, who typically have a poor prognosis, will benefit from a higher MAP target. Furthermore, the difference in MAP levels between higher and lower MAP target groups in previous trials has varied between 10 and 15 mmHg.^49^ In the largest trial to date, the higher MAP target was 77 mmHg.^15^ An even higher MAP target has been suggested for OHCA patients when optimal MAP has been determined by invasive measurements.^53^ Our aim to compare MAP targets of >65 and >85 mmHg has not been conducted before in a large clinical trial. Lastly, a meta‐analysis that included RCTs investigating blood pressure targets after cardiac arrest could only exclude a more than 25% relative risk reduction in mortality and poor functional outcome by targeting higher MAP.^14^

Other clinical trials are planned to investigate blood pressure targets after cardiac arrest in adults. The METAPHORE trial (www.clinicaltrial.gov, NCT05486884) investigates whether a MAP target >90 mmHg as compared to >65 mmHg improves functional outcome after non‐traumatic cardiac arrest in a study including 1600 participants. The NORSHOCK trial (www.clinicaltrials.gov, NCT05168462) investigates if a MAP target >55 mmHg compared to >65 mmHg has an effect on a composite outcome of mortality and severe AKI after acute myocardial infarction and cardiogenic shock. Compared to our trial, NORSHOCK differs in trial population, since non‐cardiac arrest and cardiac arrest patients are included, and it compares a lower MAP target to a “standard” MAP target. After completion, results from these trials, together with our trial, provide evidence on the optimal blood pressure strategy for post‐resuscitation care.

### 3.1 Strengths and limitations

Strengths of the MAP‐CARE trial are its large sample size, broad inclusion criteria, and the predefined detailed methodology leading to results with low risks of bias. The sample size enables the detection of small relative risk reductions or increases and comparison of intervention effects between a variety of cardiac arrest patient characteristics. This is important because individualization of MAP targets has been suggested to improve outcomes after cardiac arrest.^54^, ^55^, ^56^ Patient‐centered and clinically important outcomes together with the involvement of patient organizations, blinding of outcome assessors, prognosticators, the steering group, author group, statisticians, and the trial coordinating team represent significant strengths.

Interactions between the blood pressure, sedation, and temperature strategies, with an effect on patient‐centered outcomes, are a possibility and must be considered a limitation. This risk is implicit in all factorial trials. We have designed the study assuming that there is no interaction on the primary, secondary, and exploratory endpoints with the three strategies. Following calculation of the sample size, we have allowed for a small increment of the sample size (6.8%) to allow for loss to follow‐up and a small interaction effect. Unblinding of MAP targets is another limitation in this trial. Off‐setting blood pressure modules to facilitate blinding of MAP targets^15^ is not feasible in our trial because we will have multiple sites participating worldwide using blood pressure monitoring devices from different manufacturers.

## 4 CONCLUSION

A large clinical trial is needed to investigate the optimal blood pressure strategy in patients resuscitated from cardiac arrest. The MAP‐CARE trial aims for a large sample size and will investigate blood pressure strategies in a broad population of cardiac arrest patients. We expect that results from the MAP‐CARE trial will guide future recommendations on hemodynamic management after cardiac arrest. The MAP‐CARE trial is part of the large international factorial STEPCARE trial that will also investigate the depth of sedation and fever treatment strategies after cardiac arrest.

## AUTHOR CONTRIBUTIONS

V. H. Niemelä drafted the manuscript. All other authors contributed to the study by critically reviewing and editing the manuscript.

## FUNDING INFORMATION

The trial is funded by The Swedish Research Council, ALF‐project funding within the Swedish Health Care, The Academy of Finland, Sigrid Jusélius Foundation (Finland), Hospital District of Helsinki and Uusimaa (Finland), Medicinska Understödsföreningen Liv och Hälsa (Sweden), Medical Research Future Fund (Australia), Health Research Council of New Zealand, and the Clinical Research Programme Directorate of Health Ministry of Health and Social Security (Luxembourg).

## Supporting information


**Data S1.** Supporting Information.
